# Real-time Burn Classification using Ultrasound Imaging

**DOI:** 10.1038/s41598-020-62674-9

**Published:** 2020-04-02

**Authors:** Sangrock Lee, Rahul, Hanglin Ye, Deepak Chittajallu, Uwe Kruger, Tatiana Boyko, James K. Lukan, Andinet Enquobahrie, Jack Norfleet, Suvranu De

**Affiliations:** 1 Center for Modeling, Simulation and Imaging in Medicine, Rensselaer Polytechnic Institute, Troy, NY USA; 2 Medical Computing Team, Kitware, Inc., Clifton Park, NY USA; 3 Department of Biomedical Engineering, Rensselaer Polytechnic Institute, Troy, NY USA; 4 Department of Surgery, University at Buffalo-State University of New York, Buffalo, NY USA; 5 U.S. Army Futures Command, Combat Capabilities Development Command Soldier Center STTC, Orlando, FL USA

**Keywords:** Ultrasonography, Biomedical engineering

## Abstract

This article presents a real-time approach for classification of burn depth based on B-mode ultrasound imaging. A grey-level co-occurrence matrix (GLCM) computed from the ultrasound images of the tissue is employed to construct the textural feature set and the classification is performed using nonlinear support vector machine and kernel Fisher discriminant analysis. A leave-one-out cross-validation is used for the independent assessment of the classifiers. The model is tested for pair-wise binary classification of four burn conditions in *ex vivo* porcine skin tissue: (i) 200 °F for 10 s, (ii) 200 °F for 30 s, (iii) 450 °F for 10 s, and (iv) 450 °F for 30 s. The average classification accuracy for pairwise separation is 99% with just over 30 samples in each burn group and the average multiclass classification accuracy is 93%. The results highlight that the ultrasound imaging-based burn classification approach in conjunction with the GLCM texture features provide an accurate assessment of altered tissue characteristics with relatively moderate sample sizes, which is often the case with experimental and clinical datasets. The proposed method is shown to have the potential to assist with the real-time clinical assessment of burn degrees, particularly for discriminating between superficial and deep second degree burns, which is challenging in clinical practice.

## Introduction

The ultrasound imaging modality has emerged as a viable technique for non-invasive assessment of altered soft tissue characteristics for diagnostic purposes. Ultrasonography has been used for the assessment of burn depth in experimental model and clinical burns^1–4^. However, they lack standardization in quantifying burn depth and often limited in accuracy^5^. The burn depth determination is subjective to expert’s assessment based on A- or B-mode signals from the burn sites. In addition, our previous studies have shown that the ultrasound elastography fails to identify burn severity with acceptable accuracy when their elastic properties are not sufficiently altered to adequately contrast with the surrounding tissues^6^. To address these problems, we propose an ultrasound imaging-based machine learning approach to objectively identify altered tissue characteristics with specific application to classification of thermally treated *ex vivo* porcine skin tissue.

Burns are the most common injuries in both civilian and combat scenarios. Acute burn injury occurs in approximately 5 to 20% of combat casualties^7^. At present, in the United States, 396,974 patients are treated for nonfatal burn injuries^8^, 25,823 require hospitalization, and cost a total of $1.7 billion yearly^9^. The field of medicine has made tremendous strides in the ability to care for burn patients. Despite these leaps in the improvement of treatment of burn patients, there are still significant challenges, for example, to distinguish between various degrees of burn that require different treatment strategies. Burns traditionally are divided into three depth categories based on the degree of tissue injury: superficial (first-degree burns), partial-thickness (second-degree burns), and full-thickness (third-degree burns). Partial-thickness, or second-degree, burns are subdivided further into superficial-partial and deep-partial thickness burns. Clinical diagnosis via visual and tactile inspection is the current norm in burn depth detection, though other techniques have been developed^5^, without widespread clinical adoption. The classification accuracy to discern superficial-partial and deep-partial is between 50–80%^10–12^. This is problematic because treatment for the former is medical, whereas the latter benefits from early surgical excision. Developing an automated burn classification technique based on a tool that is readily available in all hospitals would be instrumental to improving burn care, decreasing complications and lowering costs associated with treatment.

The ultrasound imaging modality has been utilized to assess burn depth. For example, Goans *et al*.^1^ utilized ultrasound pulse-echo reflection spectra (A-mode signal) to determine burn depth in porcine skin. While the spectra of unburned porcine skin tissues show two peaks corresponding to the epidermis-dermis and dermis-subcutaneous fat interfaces, the burned tissues show one more peak at the interface between the burned and the unburned tissue. It should be noted, however, that an A-mode signal only provides very localized information that may not reveal the exact burn severity at the current location. The use of B-mode images can overcome this problem, as they cover a wider range of the skin. For example, Kalus *et al*.^2^ utilized a 5 MHz B-mode ultrasound probe to successfully evaluating burn depth in two patients: one with superficial burn and the other with a full-thickness burn. Iraniha *et al*.^3^ introduced a noncontact ultra-sonographic device to assess burn depth and achieved an accuracy of 96%. Brink *et al*.^4^ found a significant correlation (*R* = 0.9) between the ultrasound images of burn wounds and histologic sections. However, Wachtel *et al*.^13^ reported that burn depth assessment using B-mode ultrasound images alone does not provide improved accuracy compared to histological diagnosis. In addition, ultrasound image-based techniques^1,2,4^, including non-contact ultrasound^3^, are subjective to expert’s assessment of burns based on either A- or B-mode signals from the burn sites. Consequently, ultrasound image alone is rarely used to determine burn degree in practice^5^.

In our previous work^6^, we have shown that the ultrasound elastography can reliably differentiate between unburned and burned tissues, yet fails to identify burn severity with acceptable accuracy. We observed that the different burn conditions do not alter the stiffness of the tissues enough to yield a statistically significant difference in the elastic properties. Hence, burn classification based on just nonlinear elastic properties resulted in poor accuracy. In addition, the characterization of nonlinear material parameters requires the solution of an inverse optimization problem, which incurs computational cost in the range of hours, rendering it infeasible for any real-time application.

To overcome the limitations of existing techniques, we propose an ultrasound imaging-based burn classification (USBC) method in which the B-mode ultrasound images are directly used to classify burn depth. This is done by first converting the pixel intensity (grey-level) in the B-mode images into a grey-level co-occurrence matrix (GLCM) and generating statistical measures, or features, of the image texture from this matrix. The GLCM textural features are used as a variable set for a multivariate classification using the nonlinear support vector machine (SVM)^14^ and kernel Fisher discriminant analysis (KFDA)^15,16^. SVM is considered here for separating samples with two burn conditions and KFDA examines whether a group of samples with various burn conditions can be clearly separated. To assess the performance of the SVM classifiers independently, we use leave-one-out cross-validation (LOOCV).

GLCM is a 2D histogram of co-occurring greyscale intensity pairs in neighboring pixels of an image and can be used to characterize the textural properties of the image^17^. GLCM features relate to specific textural characteristics of the image such as homogeneity, contrast, and presence of organized structures within the image. Image texture information described by the GLCM is widely used in image analysis and pattern recognition in remote sensing^18^, object tracking^19^, and medical imaging for tumor detection^20–24^. Though GLCM constructed from ultrasound images has been used for detection of breast cancer^22^, prostate cancer^25^, and parotid gland injury^26^, their use for determining burn degree has not been considered. Various other methods are reported in the literature for characterizing image textures, such as grey-level size zone matrix (GLSZM)^27^, grey-level run-length matrix (GLRLM)^28^, and local binary patterns (LBP)^29^. While GLSZM is insensitive to rotation of image^27^, LBP suffers from exponentially increasing feature size with the number of neighbors^30^. The GLCM is able to identify subtle local variation of pixel intensities, which is necessary to capture altered texture characteristics for classification of burn tissues using ultrasound images. Both the GLCM and GLRLM are shown to have comparable accuracy in classification of optical images^31–33^. In this work, GLCM is used for burn classification. As we will see in the result section, this choice yields a 99% classification accuracy.

To identify altered tissue properties, various machine learning algorithms have been used. Huynen *et al*.^25^ used the k-nearest neighbor classifier (KNN) of gastric cancer metastasis in the lymph node, and Nirschl *et al*.^34^ used a deep convolutional neural network (CNN) classifier to identify abnormal heart tissue from H&E stained whole-slide image. Anantrasirichai *et al*.^35^ employed the SVM for glaucoma detection using optical coherence tomography. SVM is a binary classifier and finds an optimal hyperplane which separates two-class data with the widest margin^14^. Ultrasound images involve noise including refraction and ring-down artifacts^36^, which may yield a dataset that is linearly not separable. We therefore employ the kernel-based SVM to overcome this problem^37^.

The remainder of the paper is organized as follows: The methods and materials section describes the feature extraction, classification approach, and dataset comprised of burned *ex vivo* porcine skin tissues. This is followed by results, discussion, and conclusion sections.

## Methods and materials

### B-mode image texture analysis

The statistical estimate of the texture of B-mode images is derived from the GLCM extracted from those images. GLCM is defined as a histogram of co-occurring greyscale intensity pairs in neighboring pixels of an image^17^. The process of constructing GLCM from the greyscale images is briefly reviewed here.

An ultrasound image may be viewed as a matrix of pixel intensity *I*(*m*, *n*) that represents the grey-level value of the pixel at the (*m*, *n*)^*th*^ entry. This matrix is mapped to the GLCM whose (*i*, *j*)^*th*^ entry ${\chi }_{k,l}(i,j)$ represents how often a pixel with grey-level value *i* = *I*(*m*, *n*) occurs adjacent to a pixel with grey-level value *j* = *I*(*m* + *k*, *n* + *l*) in the B-mode image, where *k* and *l* are the offset index along the row and column, respectively. If the offset is *k* = 0 and *l* = 1, e.g., the two pixels are horizontally adjacent to each other, whereas an offset of *k* = 1 and *l* = 0 implies that they are vertically adjacent. In this study, we consider horizontally adjacent pixels to capture alternatively changing pixel intensity variation of the B-mode image. The GLCM ${\chi }_{k,l}$ can then be given as^17^,

1 $$\begin{array}{c}{\chi }_{k,l}(i,j)=\mathop{\sum }\limits_{m=1}^{M}\,\mathop{\sum }\limits_{n=1}^{N}{\rho }_{k,l}(m,\,n)\\ {\rho }_{k,l}(m,n)=\{\begin{array}{cc}1, & if\,I(m,n)=i\,and\,I(m+k,n+l)=j\\ 0, & otherwise\end{array},\end{array}$$

where ${\chi }_{k,l}\in {{\mathbb{Z}}}^{L\times L}$ and *L* is the number of quantized grey-levels, *M* is the number of rows, *N* is the number of columns in the image. In this work, the grey-level varies between 0 (black pixel) and 255 (white pixel) with an increment of 1, thus *L* = 256. The computational complexity of constructing a GLCM is of ${\mathscr{O}}(\,MN)$^38^.

### Textural feature extraction

The GLCM is used to compute second-order statistical measures (Supplementary Table S1). These measures relate to specific textural characteristics of the image such as homogeneity, contrast, and presence of organized structure within the image^17^. For example, the energy (or angular second-moment) feature is a measure of homogeneity of the image, whereas, the contrast feature measures the local variations present in an image. The correlation feature is a measure of grey-level linear-dependence in the image. Other measures characterize the complexity and nature of grey-level transitions that occur in the image. A total of nineteen (19) GLCM texture features are considered in this work^17,39,40^. These features are listed in Supplementary Information. The complexity of computation of GLCM features is ${\mathscr{O}}({L}^{2})$^38^.The interpretation of the features in Supplementary Table S1 is presented elsewhere^41^.

It is well-known that removing redundant features decreases the required size of the training data set and prevents overfitting^37^. The GLCM features listed in Supplementary Table S1 may contain correlated information, *i.e*., removing some of the features does not affect the classification accuracy. In an effort to isolate the most discriminating features we employ a sequential backward selection (SBS) method^42^.

The SBS is a top-down procedure in which the search starts with the complete set of features, discarding the feature which is least discriminatory in each stage. The least discriminatory feature is eliminated based on the classification accuracy obtained from the independent assessments using LOOCV. The algorithm terminates when removing another feature implies an increase in classification error. The SBS does not investigate all possible combinations of features, hence, the selected feature set may not be the global optimum. Nevertheless, this approach is still acceptable as it can reduce the number of features without increasing the classification error. In the results section, the effect of feature selection is investigated using KFDA. The details of the KFDA algorithm is presented in Supplementary Information.

### Classification approach

We employ a support vector machine (SVM)^14^ based approach to classify tissues with altered characteristics. SVM is a discriminative classifier defined by a separating hyperplane. It finds an optimal hyperplane which separates samples with the largest margin, where the margin is defined by the Euclidian distance to the closest training data-point from the hyperplane. The SVM is a natural binary classifier if the boundary between the two classes is linear. For data sets with nonlinear class boundaries, the SVM produces nonlinear decision boundaries by mapping the original finite-dimensional space to a much higher-dimensional space. The SVM requires “labeled” data for training. For a binary classification of tissues with altered characteristics, samples that satisfy a predefined null hypothesis is labeled as +1, while the remaining samples are identified as −1. The details of the SVM with radial basis function (RBF) kernel is presented in Supplementary Information.

**Figure 1:**
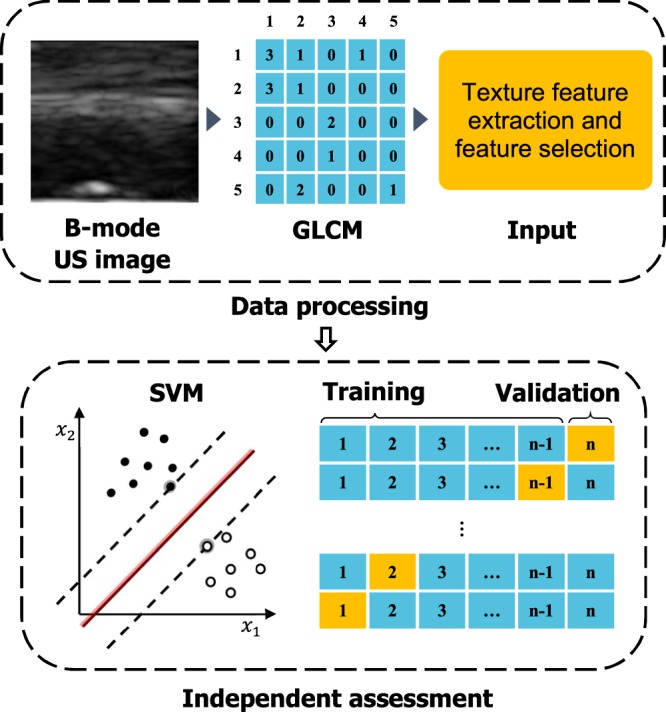
The SVM-based classification approach described in two steps: (i) extraction of GLCM texture features from the ultrasound images, and (ii) independent assessment (LOOCV) of the SVM classifier.

A LOOCV^37^ is performed for the independent assessment of the SVM classifier. The LOOCV involves splitting the sample set into a training set containing all but one observation and a validation set that includes observation left out. Since the excluded observation is not used for training, the misclassification error provides an independent estimate for the accuracy of the classifier. Unlike the validation set approach, which randomly splits the dataset into mutually exclusive training and validation sets, LOOCV does not lead to variability in the test error that may arise due to random partitioning of the dataset and insufficient number of samples in the training and validation sets, which is often the case with *ex vivo* experimental data. For every instance of the dataset, confusion matrix-based performance measures are computed and aggregated across all folds to yield an overall measure of SVM classifier accuracy^43^. The schematic of the SVM-based classification approach is presented in Fig. 1.

### Dataset

The dataset of ultrasound B-mode images of skin tissue with various degrees of the burn was obtained from *ex vivo* porcine experiments. Locally sourced porcine skin tissues were cut into 150 × 150 *mm*^2^ identical pieces. The thickness of the samples varied between 15 *mm* and 23 *mm* due to inherent morphological variations of skin and subcutaneous tissues. Samples were kept hydrated at room temperature in 1× phosphate-buffered saline solution before conducting the experiments. The samples were subjected to the desired burn conditions using a commercial griller (Cuisinart® GR-300WS Griddler Elite Grill, Conair Corporation, NJ). Ultrasound imaging was performed when the samples cooled down to the room temperature. The B-mode images of burned tissues were obtained using an Ultrasonix L40-8/12 linear array probe at 10 MHz. The imaging depth was 20–30 mm with average axial and lateral resolution of 59 microns. As we will see in the results section, this probe has sufficient spatial resolution to capture the altered texture information in the burned tissue that is needed for burn classification. The details of the experimental protocol are described elsewhere^6^.

**Figure 2:**
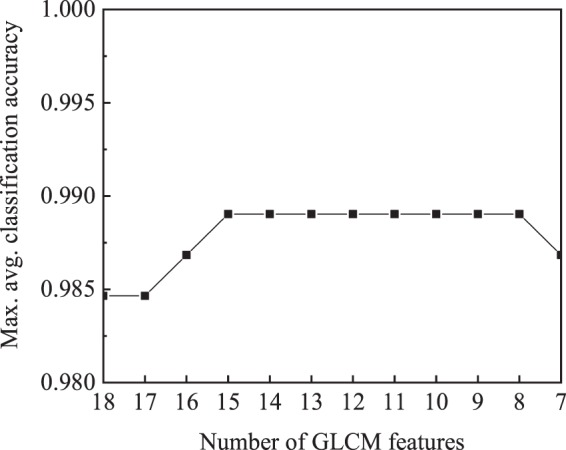
Maximum average accuracies of six pair-wise burn classifications for the given number of GLCM features.

The burn time and burn temperature are known to be inversely correlated for a given burn depth^44^. Hence, we considered four different combinations of burn time and burn temperature as surrogates of burn depth that could result in second-degree (partial thickness) and third-degree (full thickness) burns in the *in vivo* porcine tissue^45–47^. The contact burning conditions of (i) 200 °F for 10 s, (ii) 200 °F for 30 s, (iii) 450 °F for 10 s, and (iv) 450 °F for 30 s are considered in order to achieve degrees of burn ranging from superficial-partial to full-thickness burns. Specifically, burning at 200 °F for 10 s is reported to inflict a mild second-degree burn^46^ and that at 450 °F for 30 s results in a full-thickness burn^47^. Contact burning at 176 °F for 20 s is reported to create a deep-partial burn^48^, as is burning at 212 °F for 30s^49^. Since the burn condition (ii) falls in-between these two, we estimate that burning at 200 °F for 30 s could possibly result in a deep-partial burn. A specific indication for burning at 450 °F for 10 s does not exist in the literature. Interpolating the plot in Fig. 2 of^45^, we estimated that it may lead to mild third-degree burns.

Ultrasound imaging is performed on each sample in the respective burn groups. A total of 37, 33, 39, and 34 B-mode images are obtained in each of the burn groups (i), (ii), (iii), and (iv), respectively. To ensure that the accuracy of the proposed method is acceptable, given sensitivity *s*_*n*_, specificity *s*_*p*_, prevalence *P*, maximum marginal error *d*, assumed confidence level *α*, and associated standard normal critical value *z*_1−*α*/2_, the minimum sample size is given as^50^

2 $$n=\max \left(\frac{{z}_{1-\alpha /2}^{2}\,{s}_{n}(1-{s}_{n})}{{d}^{2}P},\frac{{z}_{1-\alpha /2}^{2}\,{s}_{p}(1-{s}_{p})}{{d}^{2}(1-P)}\right).$$

The positive samples are expected to vary between 40% to 50%. Hence, prevalence *P* will fall in the interval [0.4, 0.5]. For our study, we choose *P* = 0.4 because it yields the maximum number of samples based on Eq. (2) with *s*_*n*_ = *s*_*p*_ = 0.95, *d* = 0.1, *α* = 0.05, and *z*_1−*α*/2_ = 1.96. A minimum of 46 samples are required. In this work, the total number of any two sample combination is greater than 67.

## Results

### Feature selection

We begin with feature selection for burn classification using the SBS scheme as described in the methods and materials section. The feature set that yields the maximum of the average accuracies of six binary classification of four burn groups is selected within each SBS iteration. The algorithm terminates when the selected feature set decreases the maximum average accuracy. Figure 2 shows that the maximum average accuracy increases with elimination of redundant features. The removed features, such as autocorrelation and cluster prominence, do not show any consistent pattern of texture evolution with increasing burn severity. A combination of 8 GLCM features yields best maximum average accuracy. Dropping features further decreases the classification accuracy. Hence, a set of 8 GLCM features out of a total 19 features (Supplementary Table S1) is selected for the binary classification of the four burn groups. The selected feature set includes contrast, correlation, difference variance, homogeneity, information measure of correlation II, inverse difference, maximum probability, and sum entropy. The selected features show a consistent pattern of texture evolution with increasing burn severity. This aspect is further examined in the discussion section.

### Burn cluster analysis

**Figure 3:**
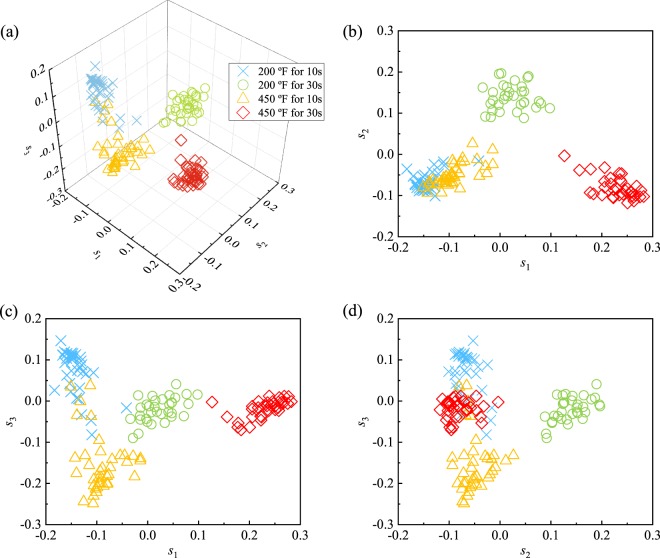
Supervised clustering of the burn groups using KFDA; *s*_1_, *s*_2_ and *s*_3_ are the discriminant analysis scores (Supplementary equation (S2.2)).

We study the efficacy of these selected features in clustering burn groups using KFDA. Figure 3(a) through 3(d) show data distribution for the selected features. In this figure, *s*_1_, *s*_2_ and *s*_3_ are the discriminant analysis scores described in the Supplementary equation (S2.2). From Fig. 3, we see that the four burn groups are distinctly different.

### Binary and multiclass classification of burn groups

**Figure 4:**
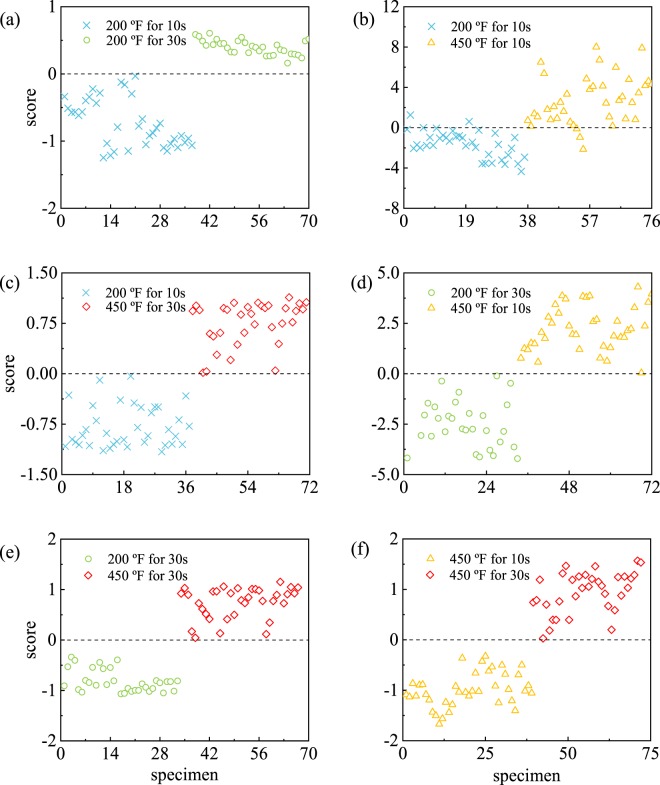
SVM score plots of the burn groups which are obtained from independent assessment of pairwise binary classification. The score on the y-axis is defined by Supplementary equation (S3.5) and the sign of the designated data determines the burn group to which the data belongs.

We study a pairwise binary classification of four burn groups using an SVM classifier with the 8 selected features. The classification performance is computed from the confusion matrix (Table 1) obtained by independent assessment using LOOCV. In Table 1, six comparisons are shown with a corresponding null hypothesis *H*_0_. The null hypotheses indicate that the data point belongs to the designated burn group. From Table 1, we see that all but one binary classification, between groups (i) and (iii), correctly identify all the specimen as belonging to the appropriate burn groups. This is further reflected in Fig. 4(a) through 4(f) that show the independently assessed SVM classification scores defined in Supplementary equation (S3.5) for the pairwise binary classification of burn groups. The SVM classification scores represent the distance from the data point to the hyperplane (*i.e*., the decision boundary). A positive score for a burn group indicates that the sample is predicted to be in that group. A negative score indicates otherwise. The corresponding performance measures, *i.e*. accuracy, sensitivity, and specificity, are presented in Table 2. Here, we see that the SVM model can classify burn groups with average accuracy, sensitivity, and specificity of 99%, 99%, and 99%, respectively. Moreover, the SVM model not only correctly classifies all the samples into two distinct burn groups, *i.e*. group (i) and (iv), but can also differentiate between burn groups that are close to each other, e.g., (i) and (ii), and (iii) and (iv). The misclassification between (i) and (iii) occurs due to the outliers observed in Fig. 3(c).

**Table 1:** Confusion matrices from the pairwise binary classification.

Burn groups	*H*_0_	Decision	True	False
200 °F for 10 s–200 °F for 30 s	200 °F for 30 s	Accept *H*_0_	33	0
		Reject *H*_0_	0	37
200 °F for 10 s–450 °F for 10 s	450 °F for 10 s	Accept *H*_0_	36	2
		Reject *H*_0_	3	35
200 °F for 10 s–450 °F for 30 s	450 °F for 30 s	Accept *H*_0_	34	0
		Reject *H*_0_	0	37
200 °F for 30 s–450 °F for 10 s	450 °F for 10 s	Accept *H*_0_	39	0
		Reject *H*_0_	0	33
200 °F for 30 s–450 °F for 30 s	450 °F for 30 s	Accept *H*_0_	34	0
		Reject *H*_0_	0	33
450 °F for 10 s–450 °F for 30 s	450 °F for 30 s	Accept *H*_0_	34	0
		Reject *H*_0_	0	39

*H*_0_ is the null hypothesis indicating the designated data belongs to the positive group in SVM classification. True and False are the true label of data and Accept *H*_0_ and Reject *H*_0_ are the decision from the classifier.

**Table 2:** Performance measure of the SVM classifier.

Burn groups	Accuracy	Sensitivity	Specificity
200 °F for 10 s–200 °F for 30 s	1.00	1.00	1.00
200 °F for 10 s–450 °F for 10 s	0.93	0.92	0.95
200 °F for 10 s–450 °F for 30 s	1.00	1.00	1.00
200 °F for 30 s–450 °F for 10 s	1.00	1.00	1.00
200 °F for 30 s–450 °F for 30 s	1.00	1.00	1.00
450 °F for 10 s–450 °F for 30 s	1.00	1.00	1.00
Average	0.99	0.99	0.99

A multiclass classification considering all four burn groups is performed using KFDA. The classification results are presented in the confusion matrix (Table 3), obtained from LOOCV. In this LOOCV scheme, each sample is assigned to one of the four classes corresponding to the four burn groups. In Table 3, the number of samples that are correctly classified are shown along the diagonal, whereas, off-diagonal components correspond to the number of misclassified samples. For example, the classification result for group (i) shows that 33 samples are correctly classified to group (i) and remaining 4 samples are misclassified to group (iii). The overall multiclass classification accuracy is estimated to be 93%, defined as correctly classified sample number divided by total sample number.

**Table 3:** Confusion matrix from the multiclass classification using KFDA.

True Label	Predicted Label				Accuracy
	200 °F for 10 s	200 °F for 30 s	450 °F for 10 s	450 °F for 30 s	
200 °F for 10 s	33	0	4	0	0.89
200 °F for 30 s	0	31	2	0	0.94
450 °F for 10 s	3	0	36	0	0.92
450 °F for 30 s	0	1	0	33	0.97

### Monte carlo simulation

We performed Monte Carlo simulations to test the generalizability of multiclass classifier. The data set is mutually-exclusively and randomly split into training and test sets, where 90% of the data is assigned to the training set and the remaining 10% to the test set. We then used the training data to train the KFDA classifier and measure the classification accuracy using the test set. After repeating this process 100 times, we obtained 92.8% average classification accuracy.

### Computational cost of pairwise classification

The average computational cost (CPU time) of computing GLCM and features (Supplementary Table S1) based on C++ implementation is 1.0 ± 9.1 × 10^−8^ *ms* and 17.6 ± 2.7 × 10^−6^ *ms*, respectively, where the implementation is repeated 1000 times. The complexity of GLCM construction is ${\mathscr{O}}(MN)$ and that of each GLCM feature is ${\mathscr{O}}({L}^{2})$. The overall cost of prediction of burn severity, stating from B-mode image, takes 19.3 ± 3.6 × 10^−6^ *ms*, which is well within the real-time computing requirements of 30 *ms*^51^. The CPU time is measured on the machine with Intel CPU 3.4 GHz.

## Discussion

The burn classification accuracy presented in the results section elucidate the efficacy of the GLCM features, extracted from ultrasound images, in accurately identifying the burn groups. In this section, we discuss how the GLCM features capture the variation in pixel intensities of ultrasound images induced by morphological changes in the tissue with burns. For this, we first analyze the characteristics of ultrasound images and GLCM features, followed by a discussion on GLCM features and their ability to capture altered characteristics of ultrasound images of burned tissue.

**Figure 5:**
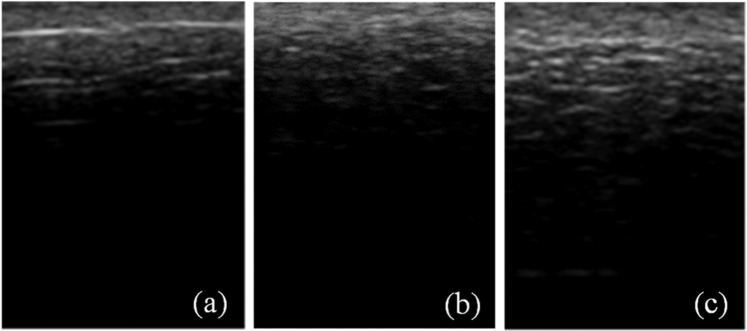
The ultrasound images of dimension 1.5 cm × 1.1 cm of porcine skin tissues showing a gradual increase in speckles with increasing burn severity starting from (**a**) unburned state to burn at 200 °F for (**b**) 10 s, (**c**) 30 s.

**Figure 6:**
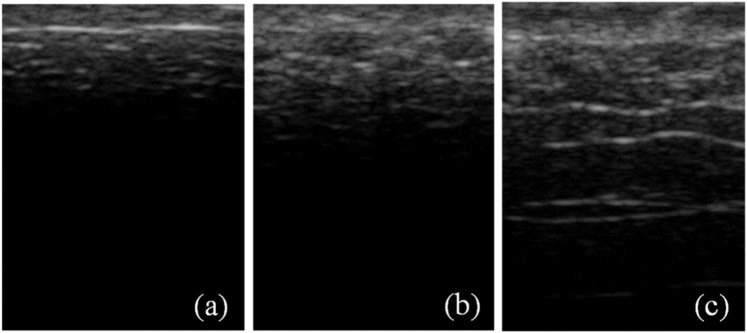
The ultrasound images of dimension 1.5 cm × 1.1 cm of porcine skin tissues showing a gradual increase in speckles with increasing burn severity starting from (**a**) unburned state to burn at 450 °F for (**b**) 10 s, (**c**) 30 s.

**Figure 7:**
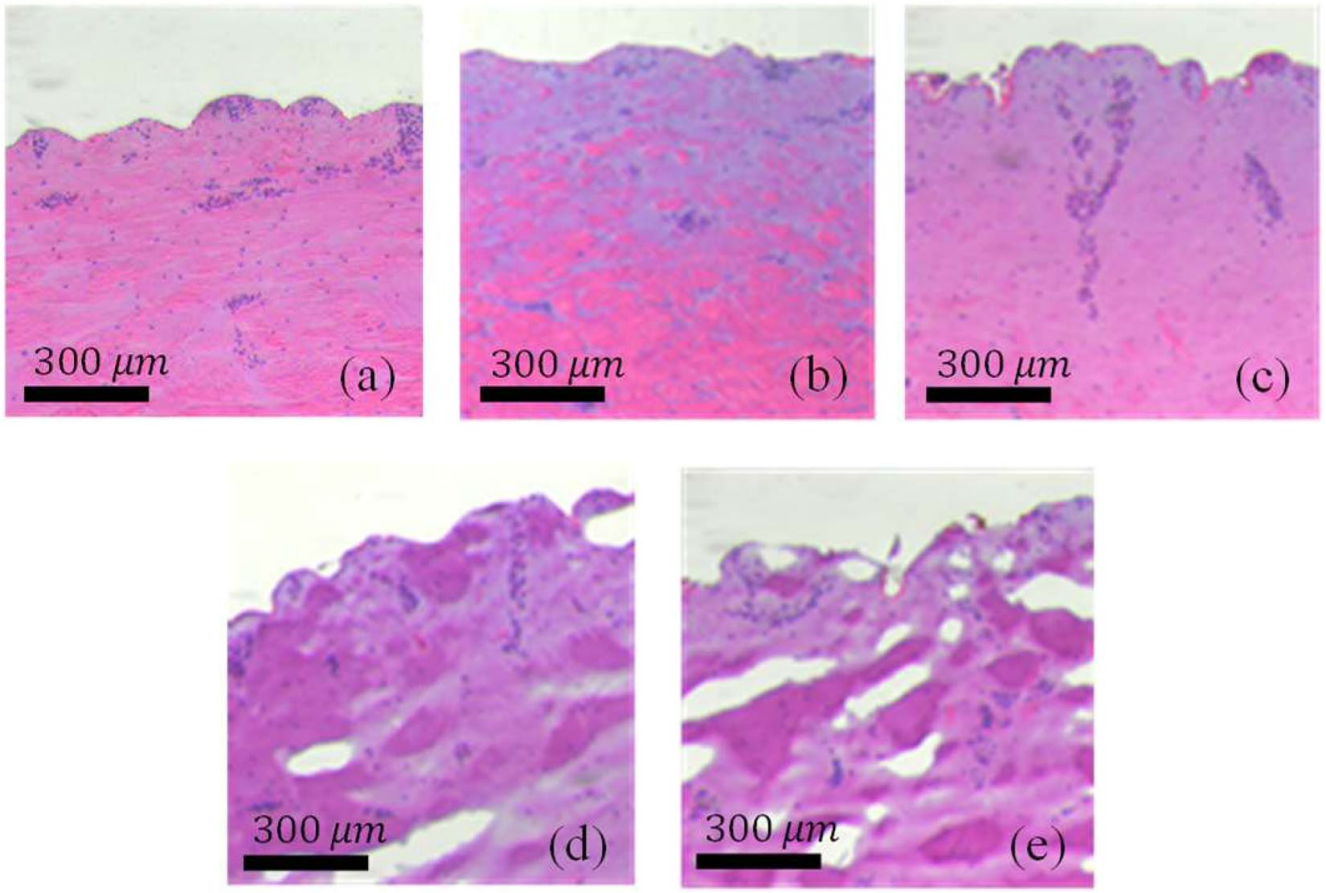
Histology of porcine skin for (**a**) unburned tissue and samples that are burned at (**b**) 200 °F for 10 s, (**c**) 200 °F for 30 s, (**d**) 450 °F for 10 s, (**e**) 450 °F for 30 s. For histology examination, a section punch biopsy is fixed in 10% formalin and embedded in paraffin, which is stained with haematoxylin and eosin (H&E) before examination under Olympus IX-71 microscope at 10× magnification.

The ultrasound images of *ex vivo* porcine skin tissue (Figs. 5 and 6) show increased speckles with increasing burn severity. The speckles consist of low-intensity pixels in the ultrasound images. From Fig. 5, we observe that the speckles not only gradually appear along the depth of the skin but also grow with increasing burn severity starting from the unburned (Fig. 5(a)) to the most severe burn at 200 °F for 30 s (Fig. 5(c)). Similar to Figs. 5 and 6 shows the growth of speckles with increasing burn severity from the unburned (Fig. 6(a)) to the most severe burn at 450 °F for 30 s (Fig. 6(c)). The speckles are a manifestation of microstructural degradation of soft hydrated tissues subjected to the applied heating. The water trapped in intra- and extra-cellular spaces evaporate by absorbing the applied heat, resulting in a large acoustic impedance difference between the vapor filled pores and the surrounding tissue^52^. Similar acoustic impedance difference can also be expected due to the altered structural integrity of the burned tissue as shown in the histology images in Fig. 7. Thermal treatment of tissues is known to change the acoustic impedance of tissue^53^. The speckles in the ultrasound images appear due to the interference of strong scattering signals from the regions of contrast acoustic impedance acting as “ultrasound contrast agents” (UCAs); thereby greatly improving the contrast-to-tissue ratio (CTR) of the ultrasound images. As we saw in the results section, this greatly enhances the accuracy, sensitivity, and specificity with which burn severity can be classified. The presence of large acoustic impedance difference between UCAs and surrounding tissue is known to greatly improve the CTR in the clinical ultrasound images^52^.

**Figure 8:**
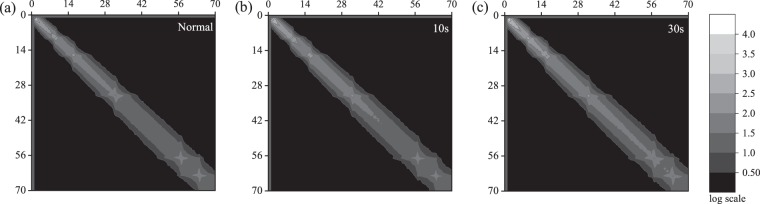
Average GLCM of the ultrasound image of *ex vivo* porcine skin tissue for (**a**) unburned state and samples burned at 200 °F for (**b**) 10 s, and (**c**) 30 s. The x- and y-axis are the row and column numbers of the GLCM and the contour is in log scale.

**Figure 9:**
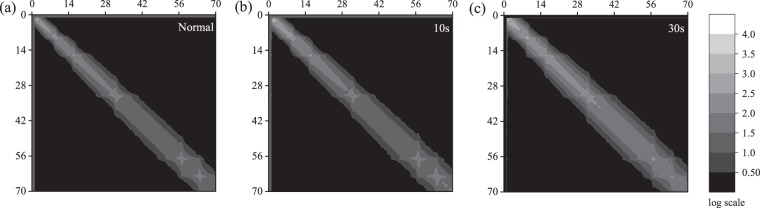
Average GLCM of the ultrasound image of *ex vivo* porcine skin tissue for (**a**) unburned state and samples burned at 450 °F for (**b**) 10 s, and (**c**) 30 s. The x- and y-axis are the row and column numbers of the GLCM and the contour is in log scale.

The textural variation in ultrasound images due to the appearance of speckles with increasing burn severity is captured in the GLCM of the ultrasound images. Figure 8(a) through 8(c) show the average GLCM of ultrasound images of unburned *ex vivo* porcine skin tissue and that of those burned at 200 °F for 10 s, and 30 s, respectively. As with Figs. 8, 9(a) through 9(c) show the average GLCM of ultrasound images of unburned *ex vivo* porcine skin tissue and that of those burned at 450 °F for 10 s, and 30 s, respectively. For illustration purposes, the arithmetic mean of GLCMs are shown for the low-intensity pixels varying from 0 to 70 corresponding to the speckles in the ultrasound images. The contours of co-occurring greyscale intensity pairs are plotted on a log scale. The left-top corner of the GLCMs represent the co-occurrence of black pixel pairs. From Figs. 8 and 9, we observe a gradual and progressive increase in the co-occurrence of grey pixel pairs with increasing burn severity. This is clearly reflected in the growing grey-colored regions along the diagonals of Fig. 8(a) through 8(c) and that of Fig. 9(a) through 9(c). The bandwidth of the grey-colored region also increases with increasing burn severity, reflecting an increase in heterogeneous pixel pairs due to the growth of speckles with burns. This clearly shows that the increase in speckles with burn severity is related to the co-occurrence of grey intensity pairs in the GLCM.

**Figure 10:**
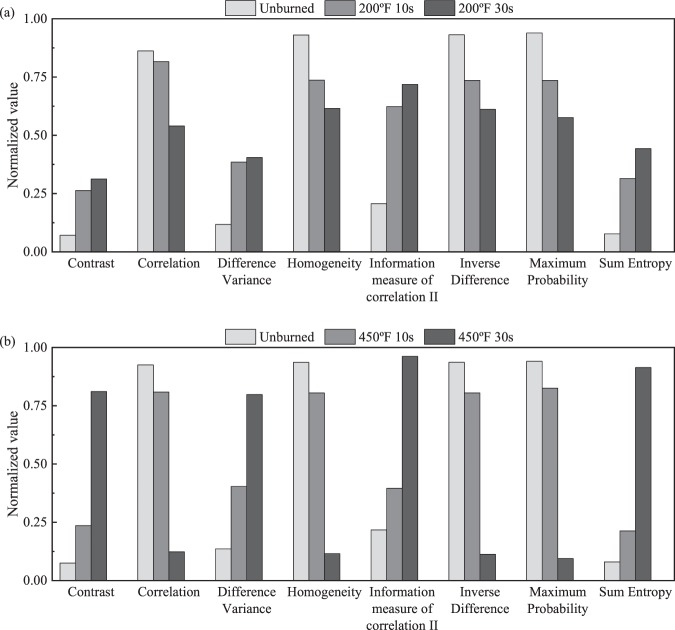
Mean values of normalized GLCM features of the burn groups (**a**) burned at 200 °F and (**b**) burned at 450 °F.

Here, we analyze the relationship between 8 selected features and textural information that is encoded in the corresponding GLCM of ultrasound images. The selected features are contrast, correlation, difference variance, homogeneity, information measure of correlation II, inverse difference, maximum probability, and sum entropy. The features are described in Supplementary Table S1. In Fig. 10(a) through 10(b), the bar chart shows the mean values of the features that are normalized to vary between 0 and 1. The vector **x** containing a single GLCM feature of all the samples is normalized by

3 $$\frac{{\bf{x}}-\,{\rm{\min }}({\bf{x}})1}{\max ({\bf{x}})-\,{\rm{\min }}({\bf{x}})}$$

where l is the vector whose components are 1’s.

The contrast feature measures local intensity variation in the ultrasound image. It is defined as a weighted sum of off-diagonal components of the GLCM. Therefore, it accounts for the co-occurrence of pixel pairs of different intensities. From Figs. 8 and 9, we know that the off-diagonal components of GLCM increase with burn severity, yielding a similar increase in the contrast feature as shown in Fig. 10.

The correlation feature measures the linear dependency of pixel intensity pairs. If the GLCM is diagonally dominant then the correlation is close to 1; otherwise, if anti-diagonal components are dominant it is close to −1. In Figs. 8 and 9, we saw that the off-diagonal components increase with burn severity, which is reflected in decreased correlation away from unity as evident from Fig. 10. The information measure of correlation II feature can be interpreted as a nonlinear counterpart of the correlation feature as it measures the nonlinear dependency of pixel intensity pairs. The mutual information *H*_*XY*2_ − *H*_*XY*_ quantifies the nonlinear dependency, which increases with the growth of speckles. Hence, unlike the correlation feature, the information measure of correlation II increases with burn severity as shown in Fig. 10.

The difference variance measures pixel intensity pair variance with respect to the diagonal line. If diagonal components are dominant, the difference variance is small. The off-diagonal component of GLCM is shown to increase with burn severity (Figs. 8–9). This, in turn, is reflected in the increasing trend of the difference variance in Fig. 10.

The homogeneity feature measures the local intensity similarity in a given ultrasound image. It is defined as a weighted sum of the pixel intensity pairs, where the weight decreases as the square of the distance from the diagonal line. It acquires high value for a diagonally dominant GLCM. On the contrary, if pixel intensity pairs are distributed far away from the diagonal, homogeneity has a lower value. As more speckles are formed, the off-diagonal components increase and, hence, homogeneity decreases. This is consistent with our observation in Fig. 10, where homogeneity is shown to decrease with burn severity. As with the homogeneity, the inverse difference feature also measures local intensity similarity in the ultrasound image. The two measures differ by the power of the weight as shown in Supplementary Table S1. Therefore, the inverse difference and homogeneity both show a decreasing trend in Fig. 10.

The maximum probability feature measures the probability of the most frequent pixel intensity pairs in the ultrasound image. With increasing burn severity, the grey-colored pixel intensity pairs spread both along the diagonal and off-diagonal due to the growth of the speckles (Figs. 8 and 9); thereby decreasing the maximum probability of occurrence of the most frequent pixel intensity pair (0, 0) in GLCM. Therefore, the maximum probability feature shows a decreasing trend in Fig. 10.

The sum entropy measures the entropy of the GLCM when viewed along the anti-diagonal direction. Entropy is a measure of the spread of pixel intensity pairs. For example, if GLCM has only one component, entropy has the lowest value, whereas, if all components are equal, then the entropy has the highest value. For unburned tissue, since most pixel intensity pairs are at the left-top corner of the GLCM, the sum entropy has a low value. With increasing burn severity, pixel intensity pairs spread along- and off-diagonal directions, leading to an increase in the sum entropy feature as shown in Fig. 10.

## Conclusion

A real-time machine learning-based approach is presented for accurate classification of burn groups using B-mode ultrasound images. Texture analysis using a grey-level co-occurrence matrix (GLCM) that is drawn from the B-mode ultrasound images is performed to extract the features for the classification of burn groups. Four different combinations of burn time and burn temperature are considered as surrogates of burn severity ranging from superficial-partial to full thickness burns. Burn classification is performed using a support vector machine (SVM) classifier with the input textural features. The independent assessment of the classifier using leave-one-out cross-validation (LOOCV) shows average accuracy, sensitivity, and specificity of 99% in binary pairwise classification and 93% in multiclass classification. The analysis of ultrasound images of *ex vivo* porcine skin tissues reveals that the speckles in the B-mode images grow with increasing burn severity. This could be due to the evaporation of intra- and extracellular tissue water, which induces a large acoustic impedance difference between vapor-filled pores and the surrounding tissues. This, along with the altered structural integrity of the tissues, creates speckles due to the interference of strong scattering signals from the regions of contrast acoustic impedance. The textural analysis further reveals that the GLCM features are able of capturing the altered characteristics of B-mode images with increasing burn severity. The proposed classification approach is shown to have the potential for assessing burn severity with relatively small datasets.

A limitation of this technique is that it uses a heuristic approach to discard least discriminatory GLCM features. A model that inherently eliminates those features that do not contribute to the classification is desirable. The next step is to apply the technique for *in vivo* burn classification which may introduce additional uncertainties associated with blood perfusion, hydration, and tissue echogenicity. These may affect the grey-scale of the ultrasound B-mode images, and hence, the accuracy of the burn classification.

## Supplementary information

Supplementary info.

## Data Availability

The datasets generated during and/or analyzed during the current study are available from the corresponding author on reasonable request.
