# Supplementary material for: Real-time Burn Classification using Ultrasound Imaging
Source: Sci Rep. 2020 Apr 2;10:5829. doi: 10.1038/s41598-020-62674-9 (PMC7118155; doi:10.1038/s41598-020-62674-9)
Supplement: Supplementary file 1 — Supplementary info. [file 41598_2020_62674_MOESM1_ESM.docx]

**Real-time Burn Classification using Ultrasound Imaging**

Sangrock Lee, Rahul, Hanglin Ye, Deepak Chittajallu, Uwe Kruger, Tatiana Boyko, James K. Lukan, Andinet Enquobahrie, Jack Norfleet and Suvranu De

**Supplementary information
S-1. Grey-level co-occurrence matrix features**

Haralick *et al*.^1^ introduced various GLCM features to characterize textures of images. Supplementary Table S.1 lists the GLCM features.

**Supplementary Table S1.** GLCM texture features^1-3^.

| **GLCM texture features** | **Formula** |
| --- | --- |
| Autocorrelation | $\Sigma_{i}\Sigma_{j}i j p(i, j)$ |
| Cluster prominence | $\Sigma_{i}\Sigma_{j}\left( i+j-\mu_{x}-\mu_{y} \right)^{4} p(i, j)$ |
| Cluster shade | $\Sigma_{i}\Sigma_{j}\left( i+j-\mu_{x}-\mu_{y} \right)^{3} p(i, j)$ |
| Contrast^*^ | $\Sigma_{i}\Sigma_{j}\left( i-j \right)^{2} p(i, j)$ |
| Correlation^*^ | $\left[ \Sigma_{i}\Sigma_{j}i j p\left( i, j \right)-\mu_{x}\mu_{y} \right]/(\sigma_{x}\sigma_{y})$ |
| Difference entropy | $-\Sigma_{i=0}^{L-1}p_{x-y}\left( i \right)\log p_{x-y}\left( i \right)$ |
| Difference variance^*^ | $\Sigma_{i=0}^{L-1}\left( i-\mu_{x-y} \right)^{2}p_{x-y}\left( i \right)$ |
| Dissimilarity | $\Sigma_{i}\Sigma_{j}\left\vert i-j \right\vert p(i, j)$ |
| Energy | $\Sigma_{i}\Sigma_{j}p\left( i, j \right)^{2}$ |
| Entropy | $-\Sigma_{i}\Sigma_{j}p\left( i,j \right)\log p\left( i,j \right)$ |
| Homogeneity^*^ | $\Sigma_{i}\Sigma_{j} p(i, j)/\left[ 1+\left( i-j \right)^{2} \right]$ |
| Information measure of correlation I | $\left( H_{XY}-H_{XY1} \right)/\max\left( H_{X},H_{Y} \right)$ |
| Information measure of correlation II^*^ | $\sqrt{1-\exp\left[ -2\left( H_{XY2}-H_{XY} \right) \right]}$ |
| Inverse difference^*^ | $\Sigma_{i}\Sigma_{j} p(i, j)/\left[ 1+\left\vert i-j \right\vert\right]$ |
| Maximum probability^*^ | $\max_{i,j} p\left( i,j \right)$ |
| Sum average | $\Sigma_{i=2}^{2L}i p_{x+y}\left( i \right)$ |
| Sum entropy^*^ | $-\Sigma_{i=2}^{2L}p_{x+y}\left( i \right)\log p_{x+y}\left( i \right)$ |
| Sum of squares | $\Sigma_{i}\Sigma_{j}\left( i-\mu_{x} \right)^{2} p(i, j)$ |
| Sum variance | $\Sigma_{i=2}^{2L}\left( i-\mu_{x+y} \right)^{2}p_{x+y}\left( i \right)$ |

where $L$ is the number of quantized grey-levels in the image; $p\left( i,j \right)$ is the $\left( i,j \right)^{th}$ entry in the normalized GLCM $\chi_{k,l}\left( i, j \right)/R$ such that $R=\Sigma_{i}\Sigma_{j}\chi_{k,l}\left( i, j \right)$ where $\chi_{k,l}\left( i, j \right)$ is defined in equation (1); $p_{x}\left( i \right)=\Sigma_{j}p\left( i,j \right)$ is the marginal probability with respect to rows and $p_{y}\left( j \right)=\Sigma_{i}p\left( i,j \right)$ is the marginal probability with respect to columns; $p_{x+y}\left( k \right)=\Sigma_{i+j=k}p\left( i,j \right)$, $k=2, 3,\ldots, 2L$ is the marginal probability with respect to anti-diagonal components and $p_{x-y}\left( l \right)=\Sigma_{\left| i-j \right|=l}p\left( i,j \right)$, $l=0,1,\ldots, L-1$ is the marginal probability with respect to diagonal components; $\mu_{x}=\Sigma_{i} i p_{x}\left( i \right)$ and $\mu_{y}=\Sigma_{j} j p_{y}\left( j \right)$, respectively, are the average pixel intensity pairs about $p_{x}$ and $p_{y}$; $\sigma_{x}^{2}=\Sigma_{i}\left( i-\mu_{x} \right)^{2}p_{x}\left( i \right)$ and $\sigma_{y}^{2}=\Sigma_{j}\left( j-\mu_{y} \right)^{2}p_{y}\left( j \right)$ are the standard deviation of pixel intensity pairs about $p_{x}$ and $p_{y}$, respectively; $\mu_{x+y}=\Sigma_{i} i p_{x+y}\left( i \right)$ and $\mu_{x-y}=\Sigma_{i} i p_{x-y}\left( i \right)$, respectively, are average pixel intensity pairs about $p_{x+y}$ and $p_{x-y}$; $H_{X}=-\Sigma_{i}p_{x}\left( i \right)\log p_{x}\left( i \right)$, $H_{Y}=-\Sigma_{j}p_{y}\left( j \right)\log p_{y}\left( j \right)$, $H_{XY}=-\Sigma_{i}\Sigma_{j}p\left( i, j \right)\log p\left( i, j \right)$, $H_{XY1}=-\Sigma_{i}\Sigma_{j}p\left( i, j \right)\log\left[ p_{x}\left( i \right)p_{y}\left( j \right) \right]$, and $H_{XY2}=-\Sigma_{i}\Sigma_{j}p_{x}\left( i \right)p_{y}\left( j \right)\log\left[ p_{x}\left( i \right)p_{y}\left( j \right) \right]$ are the entropies of corresponding probabilities, where $\log0$ is defined as 0. The features with an asterisk are the selected features using sequential backward selection.

**S-2. Kernel Fisher discriminant analysis**

Kernel Fisher discriminant analysis (KFDA) is a widely used method in machine learning, pattern recognition, and statistics as a tool of dimensionality reduction, classification and data visualization.

KFDA finds an optimal vector which maximizes the between-class variance and minimizes within-class variance by projecting data onto the optimal vector^4,5^. If the data is subdivided into $N$ classes, we can find at most $N-1$vectors, $\mathbf{w}_{1},\mathbf{w}_{2},\ldots,\mathbf{w}_{N-1},$ by solving an eigenvalue problem, associated with between-class variance and within-class variance, and the eigenmatrix $\mathbf{W}$ is given by

| $\mathbf{W}\boldsymbol{=}\left[ \mathbf{w}_{1},\mathbf{w}_{2},\ldots,\mathbf{w}_{N-1} \right]$ | (S2.1) |
| --- | --- |

where $\mathbf{w}_{1},\mathbf{w}_{2},\ldots,\mathbf{w}_{N-1}$ are arranged in descent order according to the magnitude of the eigenvalues. From equation (S2.1) the score of a new data point $s_{KDA}\left( \mathbf{x} \right)$ can be defined as

| $s_{KDA}\left( \mathbf{x} \right):=\mathbf{W}^{T}\phi\mathbf{(}\mathbf{x}\mathbf{)}=\left[ s_{1} s_{2}\ldots s_{N-1} \right]$ | (S2.2) |
| --- | --- |

where $\mathbf{x}$ is the data point and $\phi\left( \cdot\right)$ is the nonlinear mapping. Note that $s_{1}$ has the most discriminatory information among the scores, *i.e.*, $s_{1}$ maximizes the between-class variance and minimizes the within-class variance, and $s_{2}$ provides the second-best result. In this study, $N=4$ corresponds to four burn groups. We use $s_{1}$,$s_{2}$ and $s_{3}$ for data visualization in three-dimensional space.

**S-3. Support vector machine with radial basis function kernel**

In this section, SVM with RBF kernel is introduced. SVM finds an optimal hyperplane which separates data with the largest margin, where the margin is defined by the Euclidian distance to the closest point from the hyperplane. Finding the optimal hyperplane with soft margin is equivalent to solving an optimization problem given by ^6^

| $\min_{\mathbf{w},b} \frac{1}{2}\mathbf{w}^{T}\mathbf{w+}C\sum_{n=1}^{N} \zeta_{n}$  subject to: $\min_{n=1,\ldots,N} y_{n}\left( \mathbf{w}^{T}\mathbf{x}_{n}+b \right)\geq1-\zeta_{n}$ | (S3.1) |
| --- | --- |

where $\mathbf{w}$ is the normal vector of the hyperplane and $b$ is the bias, resulting in a hyperplane $\pi:\mathbf{w}^{T}\mathbf{x}+b=0$. $\mathbf{x}_{1},\mathbf{x}_{2},\ldots,\mathbf{x}_{N}$ are the training data, $y_{1}, y_{2},\ldots,y_{N}$ are the labels where $y_{i}$ can be either of +1 or -1, $\zeta_{n}\geq0$is the slack variable and $C$ is a constant which controls the shape of decision boundary such that too small $C$ results in underfitting while too large $C$ may lead to overfitting. The dual form of equation (S3.1) is given by ^6^

| $\min_{\boldsymbol{\alpha}} \frac{1}{2}\boldsymbol{\alpha}^{T}\mathbf{M}_{\mathbf{G}}\boldsymbol{\alpha-}\mathbf{1}^{T}\boldsymbol{\alpha}$  subject to: $\mathbf{y}^{T}\boldsymbol{\alpha=}0$, $0\leq\boldsymbol{\alpha}\leq\mathbf{C}$ | (S3.2) |
| --- | --- |

where $\boldsymbol{\alpha}=\left[ \alpha_{1},\alpha_{2},\ldots\alpha_{N} \right]^{T}$ is the Karush-Kuhn-Tucker (KKT) coefficient vector, $\mathbf{C}$ is the vector whose components are $C$, $\mathbf{1}$ is the vector whose components are 1’s and $\mathbf{M}_{\mathbf{G}}$ is the Gram matrix whose $(i, j)$ component $\left( \mathbf{M}_{\mathbf{G}} \right)_{ij}=y_{i}y_{j}\mathbf{x}_{i}^{T}\mathbf{x}_{j}$. In equation (S3.2), the inequality in the constraint is applied elementwise. By minimizing equation (S3.2) with respect to$\boldsymbol{\alpha}$, we obtain the classifier^6^

| $g\left( \mathbf{x} \right)=\mathrm{sign}\left( \mathbf{w}^{T}\mathbf{x}+b \right)=\mathrm{sign}\left( \sum_{n} \alpha_{n}y_{n}\mathbf{x}_{n}^{T}\mathbf{x}+b \right)$ | (S3.3) |
| --- | --- |

If a sample data is above the hyperplane $\pi$ it is classified to +1 group. In a similar way, if a sample is below the hyperplane $\pi$, it is classified to -1 group. By introducing nonlinear transformation $\phi\left( \cdot\right),$ kernel $K\left( \cdot, \cdot\right),$ the Gram matrix is transformed into $\left( \mathbf{M}_{\mathbf{G}} \right)_{ij}=y_{i}y_{j}\phi\left( \mathbf{x}_{i}^{T} \right)\phi\left( \mathbf{x}_{j} \right)=y_{i}y_{j}K\left( \mathbf{x}_{i},\mathbf{x}_{j} \right).$RBF kernel is adopted which is written by $K\left( \mathbf{x},\mathbf{y} \right)=\exp\left( -\gamma\left\| \mathbf{x}-\mathbf{y} \right\|_{2}^{2} \right)$, yielding the kernel matrix $\left( \mathbf{M}_{\mathbf{G}} \right)_{ij}=y_{i}y_{j}\exp\left( -\gamma\left\| \mathbf{x}_{i}-\mathbf{x}_{j} \right\|_{2}^{2} \right)$ where $\gamma$ is the kernel scale parameter. $C, \gamma$ are crucial factors determining classification performance so the two factors are chosen by leave-one-out cross-validation to ensure the optimal performance. $C$ and $\gamma$ used in the classification are listed in Supplimentary Table S2. The classifier $g\left( \mathbf{x} \right)$ is rewritten as

| $g\left( \mathbf{x} \right)=\mathrm{sign}\left( \sum_{n=1}^{N} \alpha_{n}y_{n}K\left( \mathbf{x}_{n},\mathbf{x} \right)+b \right)$  $b=y_{s}-\sum_{n=1}^{N} \alpha_{n}y_{n}K\left( \mathbf{x}_{n},\mathbf{x}_{s} \right)$ | (S3.4) |
| --- | --- |

where $\mathbf{x}_{s}$ is one of any support vectors, and $y_{s}$ is the corresponding label of $\mathbf{x}_{s}$. Also, we can define a score as follows:

| $s_{SVM}\left( \mathbf{x} \right)=\sum_{n=1}^{N} \alpha_{n}y_{n}K\left( \mathbf{x}_{n},\mathbf{x} \right)+b$ | (S3.5) |
| --- | --- |

where the score amounts to the distance from the data point to the optimal hyperplane. Depending on the sign of the score, the group to which the designated data belongs is determined. Time complexity of prediction is $O(n_{sv}d)$ where $n_{sv}$ is the number of support vectors and $d$ is the number of features^7^. MATLAB library was used to implement SVM. The CPU time for classification using equation (S3.4) is measured to be of the order of $O\left( 1 \right)$*μs*.

The values of hyper parameters are listed in Supplementary Table S2. The parameters $\left( C, \gamma\right)$ were chosen so that leave-one-out cross-validation can yield the minimum error in pairwise binary classification. Search range of $C$ is [0.1, 1000] and that of $\gamma$ is [0.1, 100].

**Supplementary Table S2.** KKT vector components ($C$) and kernel parameters ($\gamma$) used for burn classification.

| Burn groups | $C$ | $\gamma$ |
| --- | --- | --- |
| 200ºF for 10s - 200ºF for 30s | 0.1 | 2 |
| 200ºF for 10s - 450ºF for 10s | 100 | 10 |
| 200ºF for 10s - 450ºF for 30s | 0.6 | 1 |
| 200ºF for 30s - 450ºF for 10s | 1000 | 10 |
| 200ºF for 30s - 450ºF for 30s | 1 | 1 |
| 450ºF for 10s - 450ºF for 30s | 1 | 2 |

**References**

1 Haralick, R. M., Shanmugam, K. & Dinstein, I. Textural Features for Image Classification. *IEEE Transactions on Systems, Man, and Cybernetics* **SMC-3**, 610-621, <https://doi.org/10.1109/TSMC.1973.4309314> (1973).

2 Soh, L. K. & Tsatsoulis, C. Texture analysis of SAR sea ice imagery using gray level co-occurrence matrices. *IEEE Transactions on Geoscience and Remote Sensing* **37**, 780-795, <https://doi.org/10.1109/36.752194> (1999).

3 Clausi, D. A. An analysis of co-occurrence texture statistics as a function of grey level quantization. *Canadian Journal of Remote Sensing* **28**, 45-62, <https://doi.org/10.5589/m02-004> (2002).

4 Mika, S., Ratsch, G., Weston, J., Scholkopf, B. & Mullers, K. R. in *Neural Networks for Signal Processing IX: Proceedings of the 1999 IEEE Signal Processing Society Workshop (Cat. No.98TH8468).* 41-48.

5 Bishop, C. M., Bishop, P. N. C. C. M., Hinton, G. & Press, O. U. *Neural Networks for Pattern Recognition*. (Clarendon Press, 1995).

6 Cortes, C. & Vapnik, V. Support-Vector Networks. *Machine Learning* **20**, 273-297, <https://doi.org/10.1023/a:1022627411411> (1995).

7 Claesen, M., De Smet, F., Suykens, J. A. K. & De Moor, B. Fast Prediction with SVM Models Containing RBF Kernels. *arXiv e-prints* (2014).
